# Interaction between intestinal microbiota, inflammatory cytokines, and the expression of miR-330-3p and miR-515-5p in patients with ulcerative colitis

**DOI:** 10.5937/jomb0-54789

**Published:** 2026-02-27

**Authors:** Kaidi Qin, Chao Hu, Wenjun Li, Wulin Wang, Zilong Zhang

**Affiliations:** 1 Jingzhou Hospital Affiliated to Yangtze University, Department of Gastrointestinal, Hubei, PR. China; 2 Jingzhou Hospital Affiliated to Yangtze University, Department of Reproductive Medicine, Hubei, PR. China; 3 Jingzhou Hospital Affiliated to Yangtze University, Department of Pharmacy, Hubei, PR. China

**Keywords:** ulcerative colitis, intestinal microbiota, inflammatory cytokines, miR-330-3p, miR-515-5p, ulcerozni kolitis, crevna mikrobiota, inflamatorni citokini, miR-330-3p, miR-515-5p

## Abstract

**Background:**

Ulcerative colitis (UC) has a complex pathogenesis involving multiple factors. This study aims to explore the interplay among intestinal microbiota, serum inflammatory cytokines, and miR-330-3p, miR-515-5p in UC patients for novel treatment strategies.

**Methods:**

The study enrolled 95 healthy controls, 88 UC patients in remission, and 91 in active diseases. Assessments included clinical data, intestinal microbiota detection (using culture methods), serum cytokine measurement (using ELISA assay), and miRNA expression analysis (using RTqPCR). ROC curves evaluated diagnostic value, and multivariate logistic regression identified risk factors.

**Results:**

From the control group to UC remission and then to seizure UC, significant decreases were observed in Bifidobacterium, Lactobacillus counts, IL-10 levels, and miR-515-5p expression, while increases were noted in Enterobacteriaceae, Enterococcus counts, IL-6, TNF-<span style="color: rgb(32, 33, 34); font-family: sans-serif; font-size: 16px; background-color: rgb(248, 249, 250);">α</span> levels, and miR-330-3p expression (P&lt; 0.05). ROC curve analysis showed improved diagnostic accuracy for UC with the combination of miR-330-3p and miR-515-5p (AUC=0.899, 95% CI: 0.936 -0.978, sensitivity: 92.18%, specificity: 88.42%). miR-515-5p positively correlated with beneficial microbiota and IL-10 (r &gt; 0.5, p&lt;0.001) and negatively with harmful microbiota, IL-6, and TNF-a (r&lt; -0.5, p&lt;0.001); miR-330-3p showed opposite correlations. miR-515-5p, Bifidobacterium, Lactobacillus, and IL-10 were protective factors for UC, whereas miR-330-3p, Enterobacteriaceae, Enterococcus, IL-6, and TNF-<span style="color: rgb(32, 33, 34); font-family: sans-serif; font-size: 16px; background-color: rgb(248, 249, 250);">α</span> were risk factors.

**Conclusions:**

This study revealed interactions among intestinal microbiota, serum inflammatory cytokines, and miRNAs in UC patients, confirming the potential of miR-330-3p and miR-515-5p in UC diagnosis and assessment.

## Abstract (sr)

**Uvod:**

Ulcerozni kolitis (UC) ima složenu patogenezu koja uključuje vise faktora. Ova studija ima za cilj da ispita međudejstvo između crevne mikrobiote, serumskih inflamatornih citokina i miR-330-3p, miR-515-5p kod pacijenata sa UC radi pronalaženja novih strategija lečenja.

**Metode:**

Studija je obuhvatila 95 zdravih ispitanika, 88 pacijenata sa UC u remisiji i 91 pacijenta sa aktivnom bolešću. Procene su uključivale kliničke podatke, detekciju crevne mikrobiote (kultivacionim metodama), merenje nivoa citokina u serumu (ELISA testom) i analizu ekspresije miRNA (RT-qPCR metodom). ROC krive su korisćene za procenu dijagnostičke vrednosti, a multivarijantna logistička regresija za identifikaciju faktora rizika.

**Rezultati:**

Od kontrolne grupe, preko remisije UC, do aktivnog UC, primećeno je značajno smanjenje broja Bifidobacterium i Lactobacillus bakterija, nivoa IL-10 i ekspresije miR-515-5p, dok je uočeno povećanje broja Enterobacteriaceae i Enterococcus bakterija, nivoa IL-6, TNF-<span style="color: rgb(32, 33, 34); font-family: sans-serif; font-size: 16px; background-color: rgb(248, 249, 250);">α</span> i ekspresije miR-330-3p (P&lt; 0.05). Analiza ROC krive pokazala je poboljsanu dijagnostičku tačnost za UC kombinaciom miR-330-3p i miR-515-5p (A U C = 0,899, 95% CI: 0,936 -0,978, osetljivost: 92,18% , specifičnost: 88,42% ). miR-515-5p je bio pozitivno korelisan sa korisnom mikrobiotom i IL-10 (r&gt; 0,5, p&lt; 0,001), a negativno sa stetnom mikrobiotom, IL-6 i TNF-a (r&lt;-0,5, p&lt; 0,001); miR-330-3p je pokazao suprotne korelacije. miR-515-5p, Bifidobacterium, Lactobacillus i IL-10 identifikovani su kao zaštitni faktori za UC, dok su miR-330-3p, Enterobacteriaceae, Enterococcus, IL-6 i TNF-<span style="color: rgb(32, 33, 34); font-family: sans-serif; font-size: 16px; background-color: rgb(248, 249, 250);">α</span> predstavljali faktore rizika.

**Zaključak:**

Ova studija je otkrila interakcije između crevne mikrobiote, serumskih inflamatornih citokina i miRNA kod pacijenata sa UC, potvrdivši potencijal miR-330-3p i miR-515-5p u dijagnostici i proceni UC.

## Introduction

Ulcerative colitis (UC) is a persistent, nonspecific inflammatory disorder. It primarily impacts the rectum and colon and is marked by persistent and widespread inflammatory alterations in the colorectal mucosa. UC has a high recurrence rate and is associated with malignancy. For this reason, the World Health Organization classifies it as a refractory condition [1][2]. The gut microbiota, a vast microecosystem within the human body, plays a vital role in maintaining intestinal health. In individuals with UC, disruptions in the gut microbiota balance not only weaken the intestinal barrier but also promote persistent inflammatory responses [3][4].

Furthermore, inflammatory cytokines are key mediators of intestinal inflammation, with their expression levels directly reflecting disease activity [5]. Abnormal expression of several inflammatory cytokines including interleukin-6 (IL-6), interleukin-10 (IL-10) and tumour necrosis factor-alpha (TNF-α) - has been identified as a critical driver of sustained intestinal inflammation during UC progression [6][7][8]. Recent studies increasingly highlight the pathophysiological significance of microRNAs (miRNAs) in UC. Emerging evidence suggests dynamic interactions between miRNAs, gut microbiota, and inflammatory pathways [9][10]. Longo et al. [11] have highlighted a correlation between the abnormal expression of miR-122 and miR-145, alterations in gut microbiota, and changes in inflammatory cytokines, all of which are crucial in the advancement of intestinal inflammation. The miR-181 family has been identified as being modulated by gut microbiota, playing a mediatory role in inflammatory responses [12]. Therefore, identifying novel miRNAs and elucidating their interactions with the gut microbiota and inflammation is crucial for revealing the aetiology and pathogenesis of UC and for identifying new therapeutic targets and approaches.

In our prior research, the abnormal expressions of miR-515-5p and miR-330-3p in the faeces of patients with UC have garnered attention. In gut microbiota studies, Li et al. [13] suggested that miR-515-5p could potentially influence the intestinal microenvironment by affecting bacterial growth. Indeed, miR-515-5p plays a pivotal regulatory role in various diseases, such as facilitating apoptosis in colorectal cancer cells [14], modulating inflammatory responses in osteoarthritis [15], and impacting vascular cell damage in atherosclerosis [16]. However, in the context of UC, the link between miR-515-5p and the gut microbiota remains to be further explored. As for miR-330-3p, although no direct reports are linking it to the gut microbiome, Chen et al. have demonstrated its significant correlation with UC [17]. As a hot topic in cancer research [18], miR-330-3p not only participates in the glycolysis process to regulate colorectal cancer [19][20] but also plays a crucial role in immune regulation [21]. Therefore, a thorough investigation into the interaction mechanisms between miR-515-5p and miR-330-3p with the gut microbiota and their impact on the development of UC holds significant theoretical and clinical value.

In summary, this study aims to systematically investigate the interplay among miR-515-5p, miR-330-3p expression levels, gut microbiota composition, and inflammatory cytokine levels in UC patients by examining their intestinal tissues. The ultimate goal is to provide theoretical insights and experimental support for elucidating the pathogenesis of UC and identifying novel therapeutic targets.

## Materials and methods

### Study population

The retrospective case-control study enrolled 179 patients diagnosed with UC based on clinical and colonoscopy evaluations at Jingzhou Hospital, affiliated with Yangtze University. These patients were stratified into the remission group (Sutherland Disease Activity Index (DAI)≤2, n = 88) and the seizure group (DAI>2, n = 91) groups based on standardised scoring [22]. The control group comprised 95 age- and sex-matched healthy individuals recruited through community health screenings (HC) during the same period. Matching criteria included: 1) Age (±3 years) and sex (1:1 ratio) matching to UC patients; 2) BMI within ±2 kg/m^2^ of the case group mean; 3) Residence in the same geographic region (Jingzhou District) for ≥5 years. All participants underwent rigorous screening procedures. Exclusion criteria included (applied to all groups): 1) autoimmune diseases; 2) coexistence of diabetes and severe infections; 3) presence of severe hepatic, renal, cardiovascular, cerebrovascular, haematological, or malignant diseases; 4) pregnancy or lactation; and 5) concurrent intestinal infections or use of antibiotics, probiotics, or yoghurt within the past 2 months.

Informed consent was obtained from all participants prior to enrolment. The procedures used in this study adhere to the tenets of the Declaration of Helsinki, and the study protocol was thoroughly reviewed and accepted by the Jingzhou Hospital, which is affiliated with the Yangtze University Ethics Committee, to ensure the protection of the rights of all participants.

### Intestinal flora detection

Fresh faecal samples were obtained from all subjects and placed in sterile containers. During sample processing, 1 gram of faecal material was mixed uniformly with 9 millilitres of sterile 0.9% NaCl solution and stirred at 300 rpm for 1 minute. Subsequently, a 10-fold serial dilution was performed up to 10^-8^. For cultivation and identification, selective media tailored to the growth needs of different bacterial groups were utilised: Bifidobacterium was cultivated in a modified MRS medium (enhanced with specific growth factors) under rigorous anaerobic conditions at 37°C for 48 hours. Meanwhile, Lactobacillus was grown on MRS medium under facultative anaerobic conditions, also maintained at 37°C, for 48 hours. Enterobacteriaceae and Enterococcus were respectively grown on eosin-methylene blue medium and enterococcus-selective medium under aerobic conditions at 37°C for 18-24 hours. Ultimately, the results were expressed as the logarithm of colony-forming units per gram of wet faeces (log CFU/g) to quantify the bacterial populations.

### Detection of IL-10, IL-6, and TNF-α levels in serum

Collect 5 mL of fasting venous blood from patients. After standing for 30 minutes, centrifuge at 3000 rpm for 15 minutes. Take the upper serum for later use. Detect the levels of serum IL-10, IL-6, and TNF-α by enzyme-linked immunosorbent assay (ELISA kit, Yita Biotechnology Co., Ltd., Beijing, China). Strictly follow the instructions of the kit manual. First, dilute the standard solution into five gradients. Take 50 μL of each gradient and add it to the designated well. Incubate at 37°C under a sealed plate membrane for 30 minutes, then wash with PBS. After drying, add equal volumes of chromogenic agent A and chromogenic agent B in sequence. Incubate in a dark room at 37°C for 10 minutes for colour development. 50 μL of termination solution was dispensed into each well, followed by thorough mixing. After the reaction is terminated, a spectrophotometer (Thermo Fisher Scientific, Waltham, MA, USA) will be utilised to measure the absorbance at 450 nm. Subsequently, the concentrations of the respective factors are calculated.

### Detection of miR-515-5p and miR-330-3p expression in serum

Total RNA was extracted from serum samples using the Trizol kit (Tiangen Biotech, Beijing, China). Subsequently, the concentration of the extracted RNA was measured using a Nanodrop 2000c ultramicro spectrophotometer (NanoDrop Technologies, USA). Following this, the total RNA was reverse transcribed into cDNA utilising the PrimeScript RT Enzyme Mix I kit (TaKaRa, Japan). The components of the reverse transcription reaction mixture included 2 μL of 5x gDNA Buffer, 2 μL of 10x King RT Buffer, 1 μL of FastKing RT Enzyme Mix, 2 μL of FQ-RT Primer Mix, 2 μg of RNA, and RNase-Free ddH_2_O, which was added to achieve a total volume of 20 μL. The reaction proceeded at 42°C for 15 minutes, followed by a heating step at 95°C for 3 minutes. Subsequently, the resultant cDNA served as the template for quantitative polymerase chain reaction (qPCR), adhering to the guidelines provided by the SYBR Green qPCR Super Mix kit (Invitrogen, USA). During the data analysis, U6 was chosen as the reference gene, and the 2-^ΔΔ^Ct method was employed to determine the relative expression levels of the miR-515-5p and miR-330-3p.

### Statistical analysis

SPSS software, version 21.0, was used for statistical analyses. Continuous variables that followed a normal distribution were reported using the mean, accompanied by its standard deviation (x±s). For comparisons involving two groups, the independent samples t-test was applied, whereas for comparisons encompassing multiple groups, one-way ANOVA was utilised for analysis. For categorical variables, the chi-square (χ^2^) test was employed. The association between serum miR-515-5p and miR-330-3p expression levels and gut microbiota abundance and inflammatory cytokine levels was assessed using Pearson's correlation coefficient. Statistical significance was defined as a P-value of less than 0.05.

## Results

### Expression and diagnostic value of serum miR-330-3p and miR-515-5p in UC patients

**Figure 1:**
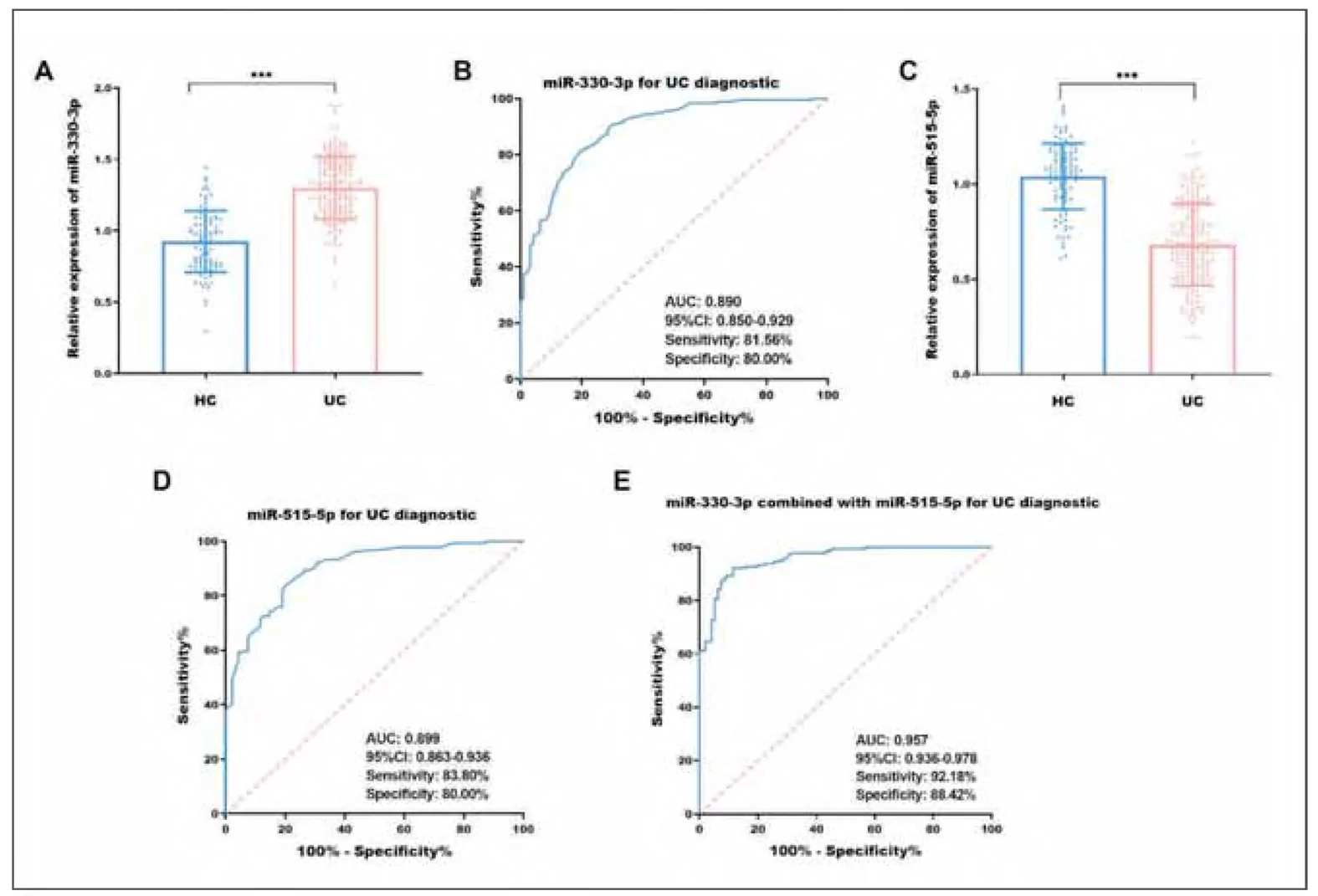
Expression and diagnostic value of serum miR-330-3p and miR-515-5p in UC.<br>A, The expression of miR-330-3p in the serum of UC patients was higher than that in the HC group. B, Diagnostic value of miR-330-3p for UC. C, The expression of miR-515-5p in the serum of UC patients was lower than that in the HC group. D, Diagnostic value of miR-515-5p for UC. E, Diagnostic value of miR-330-3p combined with miR-515-5p for UC.

The baseline clinical characteristics (age, sex, BMI) showed no significant differences between HC and UC patients (P>0.05; Table 1), confirming comparability between groups. Serum miR-330-3p expression was markedly elevated in UC patients versus HC (P<0.001, Figure 1A), with ROC analysis demonstrating strong diagnostic potential (AUC=0.890, sensitivity 81.56%, specificity 80.00%, Figure 1B). Conversely, miR-515-5p levels were significantly reduced in UC (P<0.001, Figure 1C), showing comparable diagnosis accuracy (AUC=0.899, sensitivity 83.80%, specificity 80.00%, Figure 1D). Notably, combining both miRNAs achieved superior diagnostic performance (AUC=0.957, sensitivity 92.18%, specificity 88.42%, Figure 1E).

**Table 1:** Comparison of clinical baseline data between healthy controls and ulcerative colitis patients.

Parameter	HC	UC	P value
Age (year)	38.83±11.28	41.08±11.22	0.116
Gender<br>(male/female)	49/46	89/90	0.770
BMI (kg/m^2^)	24.43±1.97	24.20±2.01	0.356

### Microbiota, cytokine, and miRNA profiling by disease severity

Stratification by disease activity (Table 2) revealed significant microbiota shifts: Bifidobacterium and Lactobacillus decreased progressively from HC to remission to active UC (P<0.05), while Enterobacteriaceae and Enterococcus increased (P<0.05). Serum IL-6 and TNF-α followed a similar trend (active > remission > HC), inversely correlating with IL-10 (P<0.05). miR-330-3p expression increased with disease severity (P<0.05), whereas miR-515-5p decreased (P<0.05).

**Table 2:** Comparative analysis of gut microbiota, inflammatory cytokines, miR-330-3p, and miR-515-5p levels between healthy controls and UC patients with varying disease. ‘ Comparison with HC group P<0.05. ^#^ Comparison with remission group P<0.05.

Parameter	HC (n = 95)	Remission (n= 88)	Seizure (n = 91)
Bifidobacterium (lg CFU/g)	10.2±1.13	8.47±1.38‘	4.81±0.85‘^#^
Lactobacillus (lg CFU/g)	9.94±0.91	7.68±0.9*	4.2±0.6*^#^
Enterobacteriaceae (lg CFU/g)	7.81±1.15	10.01±0.78*	12.62±0.87‘^#^
Enterococcus (lg CFU/g)	7.16±0.39	8.18±0.44*	8.89±0.49‘^#^
IL-6 (ng/L)	49.56±5.45	87.27±12.53‘	99.87±18.07‘^#^
IL-10 (ng/L)	58.05±8.02	36.89±8.55‘	27.16±8.58‘^#^
TNF-α (ng/L)	104.04±17.82	147.41±16.9‘	169.45±13.91‘^#^
miR-330-3p	0.92±0.22	1.19±0.18‘	1.41±0.20‘^#^
miR-515-5p	1.04±0.17	0.79±0.19‘	0.58±0.19‘^#^

### Correlation between serum inflammatory cytokines and gut microbiota

Analysis of the link between serum inflammatory cytokines and gut microbiota abundance in UC patients reveals the following: Beneficial bacteria (Bifidobacterium, Lactobacillus) inversely correlated with IL-6 and TNF-α (r≤-0.5, p<0.05), but positively with IL-10 (r≥0.52, p<0.05). Pathogenic bacteria (Enterobacteriaceae, Enterococcus) showed opposing trends (Table 3).

**Table 3:** Correlation between inflammatory factors and intestinal flora.

Variable	IL-6 (ng/L)		IL-10 (ng/L)		TNF-α (ng/L)	
	r	p	r	p	r	p
Bifidobacterium (lg CFU/g)	-0.675	<0.0001	0.578	<0.0001	-0.625	<0.0001
Lactobacillus (lg CFU/g)	-0.676	<0.0001	0.551	<0.0001	-0.635	<0.0001
Enterobacteriaceae (lg CFU/g)	0.705	<0.0001	-0.625	<0.0001	0.715	<0.0001
Enterococcus (lg CFU/g)	0.604	<0.0001	-0.650	<0.0001	0.557	<0.0001

### Correlation between serum miR-330-3p and miR-515-5p with gut microbiota

miR-515-5p positively associated with beneficial bacteria (r≥0.5, p<0.05) and inversely with pathogens (r≤-0.5, p<0.05). miR-330-3p displayed opposing trends: negative correlations with probiotics (r≤-0.5, p<0.05) and positive links to pathogens (r≥0.5, p<0.05) (Table 4).

**Table 4:** Correlation of serum miR-330-3p and miR-519-5p with intestinal flora.

Variable	miR-515-5p		miR-330-3p	
	r	p	r	p
Bifidobacterium (lg CFU/g)	0.557	<0.0001	-0.570	<0.0001
Lactobacillus (lg CFU/g)	0.556	<0.0001	-0.601	<0.0001
Enterobacteriaceae (lg CFU/g)	-0.612	<0.0001	0.672	<0.0001
Enterococcus (lg CFU/g)	-0.748	<0.0001	0.685	<0.0001

### Correlation between serum miR-330-3p, miR-515-5p, and inflammatory cytokines

miR-515-5p inversely correlated with IL-6/TNF-α (r≤-0.5, p < 0.05) and positively with IL-10 (r≥0.5, p<0.05). miR-330-3p synergized with pro-inflammatory cytokines (IL-6, TNF-α) (r≥0.5, p<0.05) while antagonizing IL-10 (r≤-0.5, p<0.05) (Table 5).

**Table 5:** Correlation of serum miR-330-3p and miR-519-5p with inflammatory factors.

Variable	miR-515-5p		miR-330-3p	
	r	p	r	p
IL-6 (ng/L)	-0.580	<0.0001	0.555	<0.0001
IL-10 (ng/L)	0.639	<0.0001	-0.669	<0.0001
TNF-α (ng/L)	-0.538	<0.0001	0.638	<0.0001

### Multivariate logistic regression analysis of risk factors for UC

UC Risk Determinants: Elevated miR-515-5p expression and increased Bifidobacterium abundance were identified as independent protective factors against UC development, with Lactobacillus and IL-10 demonstrating similar protective patterns (OR<1, P<0.05). Conversely, miR-330-3p overexpression, Enterobacteriaceae/Enterococcus overgrowth, and elevated IL-6/TNF-α levels emerged as significant risk contributors (OR>1, P<0.05; Table 6).

**Table 6:** Logistic regression analysis of risk factors for UC.

Variable	OR	95%CI	P
miR-330-3p	4.234	2.288-7.834	<0.001
miR-515-5p	0.388	0.216-0.698	0.002
Bifidobacterium (lg CFU/g)	0.527	0.294-0.943	0.031
Lactobacillus (lg CFU/g)	0.523	0.291-0.937	0.029
Enterobacteriaceae (lg CFU/g)	1.897	1.052-3.423	0.033
Enterococcus (lg CFU/g)	1.841	1.017-3.333	0.044
IL-6 (ng/L)	2.112	1.164-3.832	0.014
IL-10 (ng/L)	0.513	0.283-0.928	0.027
TNF-α (ng/L)	1.845	1.012-3.365	0.046

Disease Severity Correlates: Higher miR-515-5p and Bifidobacterium levels were inversely associated with disease progression, paralleled by protective trends in Lactobacillus and IL-10 (OR<1, P<0.05). Notably, miR-330-3p upregulation, pathogenic bacterial expansion, and pro-inflammatory cytokines exhibited dose-dependent relationships with clinical severity (OR>1, P<0.05; Table 7).

**Table 7:** Logistic regression analysis of risk factors for UC seizure.

Variable	OR	95%CI	P
miR-330-3p	4.306	1.904-9.739	<0.001
miR-515-5p	0.280	0.125-0.628	0.002
Bifidobacterium (lg CFU/g)	0.422	0.187-0.952	0.038
Lactobacillus (lg CFU/g)	0.405	0.182-0.899	0.026
Enterobacteriaceae (lg CFU/g)	2.412	1.063-5.473	0.035
Enterococcus (lg CFU/g)	3.097	1.321-7.26	0.009
IL-6 (ng/L)	2.804	1.238-6.352	0.013
IL-10 (ng/L)	0.344	0.152-0.776	0.010
TNF-α (ng/L)	2.246	1.013-4.977	0.046

## Discussion

This study explores in depth the complex relationships among gut microbiota (specifically Bifidobacterium, Lactobacillus, Enterobacteriaceae, and Enterococcus), inflammatory cytokines (IL-10, IL-6, and TNF-α), and two key miRNAs (miR-515-5p and miR-330-3p) in the pathogenesis of UC. Through detailed analysis, it uncovers an interconnected yet distinct network of interactions among these molecules, providing new insights into the pathophysiological processes of UC.

Maintaining a balanced gut microbiota is crucial for intestinal health [23]. The study reveals that Bifidobacterium and Lactobacillus, acting as probiotics, exhibit a negative correlation with the severity of inflammation in UC patients. These bacteria exert protective effects on UC through various mechanisms, including modulating intestinal immunity, inhibiting the growth of harmful bacteria, and enhancing intestinal barrier integrity [24]. For instance, Lactobacillus johnsonii activates macrophages via the TLR1/2-STAT3 pathway to promote IL-10 secretion, thereby alleviating colonic inflammation [25]. Additionally, studies indicate that Bifidobacterium can induce IL-10 production in the colon, creating a localised anti-inflammatory microenvironment [26][27]. These observations align with our data, showing a positive correlation between Bifidobacterium abundance and IL-10 levels, reinforcing the anti-inflammatory role of this cytokine in UC. Conversely, pathogenic bacteria like Enterobacteriaceae and Enterococcus drive inflammation through bidirectional interactions with cytokines. Enterococcus species, such as Enterococcus faecalis, induce IL-6 and TNF-α production via TLR2/NF-κB signalling triggered by bacterial cell wall components (e.g., lipoteichoic acid) [28]. These harmful bacteria may exacerbate the pathological process of UC by producing deleterious substances, disrupting the intestinal barrier, or triggering immune responses [29][30]. Research has shown that Enterococcus exacerbates inflammation by secreting cytotoxins and upregulating pro-inflammatory cytokines (IL-6, TNF-α) [31]. This cytokine surge disrupts epithelial tight junctions (e.g., downregulating zonulin-1), increasing permeability and bacterial translocation. Elevated IL-6 and TNF-α further alter the microbial niche: high TNF-α levels reduce intestinal oxygen tension, favouring facultative anaerobes like Enterobacteriaceae and Enterococcus [32][33]. This creates a self-reinforcing loop where dysbiosis perpetuates inflammation, and inflammation exacerbates microbial imbalance. Our study quantifies this crosstalk through significant correlations: Enterobacteriaceae/Enterococcus positively correlate with IL-6 and TNF-α, while Bifidobacterium/Lactobacillus inversely correlate with these cytokines. Multivariate analysis confirms Enterobacteriaceae and Enterococcus as independent risk factors, with IL-6 and TNF-α exacerbating disease progression. These findings highlight the microbiota-cytokine axis as a central driver of UC pathogenesis.

As crucial regulatory molecules, miRNAs have been shown in multiple studies to interact with the gut microbiota in UC [34][35][36]. This study's findings reveal a significant downregulation of miR-515-5p expression in UC patients. Further analysis demonstrates a positive link between miR-515-5p expression and beneficial bacteria, while a negative correlation is observed with harmful bacteria. In vitro experiments conducted by Ji et al. [37] also corroborate this, showing that miR-515-5p can interfere with the growth of harmful bacteria such as Fusobacterium nucleatum and Escherichia coli. This discovery indicates that miR-515-5p may exert a protective effect on UC by modulating the balance of intestinal flora. Furthermore, this study found that in UC patients, the expression of miR-330-3p positively correlates with harmful bacteria and negatively correlates with beneficial bacteria. This preliminary finding indicates a certain correlation between intestinal flora and miR-330-3p.

Additionally, miR-515-5p has been implicated in immune-inflammatory responses. Studies suggest that miR-515-5p can inhibit the immune evasion of colorectal cancer cells [38]. Cai et al. [39] found that miR-515-5p suppresses the expression of TLR4, subsequently inhibiting the activation of the MyD88/NF-κB pathway, thereby alleviating inflammatory responses in chondrocytes. Furthermore, this study confirms a significant correlation between miR-515-5p and inflammatory cytokines, further supporting the potential role of miR-515-5p in reducing intestinal inflammation by inhibiting the activation of intestinal immune cells and the release of inflammatory mediators. In contrast, the expression pattern of miR-330-3p is opposite to that of miR-515-5p. Chen et al. [17] reported that miR-330-3p is upregulated in UC and further indicated that inhibiting miR-330-3p expression can prevent dexone-induced cellular inflammatory responses. Additionally, Li et al.'s research showed that miR-330-3p downregulation can ameliorate inflammatory responses in lung cells by targeting HIF1a [40]. This study also confirms the positive correlation between miR-330-3p and inflammatory responses, reinforcing its regulatory role in intestinal inflammation.

While this study identifies significant correlations among gut microbiota, cytokines, and miRNAs in UC, several limitations require addressing. The observational design precludes causal inference, necessitating functional validation via miRNA perturbation experiments (e.g., overexpression/knockdown) to confirm direct regulatory relationships. Additionally, preclinical findings require clinical validation to ensure translational relevance and the focus on four bacterial taxa and two miRNAs limits understanding of complex host-microbiota-metabolite interactions. To address these gaps, future research should use dual-luciferase assays to validate miRNA targeting of bacterial genes (e.g., E. coli flagellin), integrate longitudinal multiomics datasets (metagenomics, transcriptomics, metabolomics) to map dynamic microbiota-miRNA-metabolite networks and conduct multicenter trials to validate miR-515-5p and miR-330-3p as diagnostic/prognostic biomarkers while exploring miRNA-based therapeutics. Randomised controlled trials evaluating probiotic-miRNA modulator combinations could also restore microbiota balance and reduce inflammation. These approaches will deepen mechanistic insights and accelerate precision interventions targeting the microbiota-cytokine-miRNA axis in UC.

In summary, the study uncovers the potential of miR-515-5p and miR-330-3p as diagnostic biomarkers for UC. It demonstrates that abnormal expression of these miRNAs in UC patients is linked to inflammatory cytokine dysregulation and gut microbiota imbalance. There exists a complex interplay among gut microbiota, inflammatory cytokines, and miRNAs. Further research into these molecular interactions may lead to novel treatment strategies for UC, ultimately enhancing patients' quality of life and reducing healthcare system burdens.

## Dodatak

### Funding

This research was funded by the Natural Science Foundation of Hubei Province (2022CFB088).

### Authors' contribution

Kaidi Qin and Chao Hu should be considered joint first authors.

### Conflict of interest statement

All the authors declare that they have no conflict of interest in this work.
